# Short O-GlcNAcase Is Targeted to the Mitochondria and Regulates Mitochondrial Reactive Oxygen Species Level

**DOI:** 10.3390/cells11111827

**Published:** 2022-06-02

**Authors:** Patrick Pagesy, Abdelouhab Bouaboud, Zhihao Feng, Philippe Hulin, Tarik Issad

**Affiliations:** 1 Université Paris Cité, CNRS, INSERM, Institut Cochin, 75014 Paris, France; patrick.pagesy@inserm.fr (P.P.); abdelouhab.bouaboud@inserm.fr (A.B.); zhihao.feng@inserm.fr (Z.F.); 2 Université de Nantes, CHU Nantes, Inserm, CNRS, SFR Santé, INSERM UMS 016, CNRS UMS 3556, 44000 Nantes, France; philippe.hulin@univ-nantes.fr

**Keywords:** O-GlcNAcylation, O-linked N-acetylglucosamine transferase, O-GlcNAc transferase, O-linked N-acetylglucosaminidase, O-GlcNAcase, *MGEA5*, mitochondria, reactive oxygen species, hydrogen peroxide, oxidative stress

## Abstract

O-GlcNAcylation is a reversible post-translational modification involved in the regulation of cytosolic, nuclear, and mitochondrial proteins. Only two enzymes, OGT (O-GlcNAc transferase) and OGA (O-GlcNAcase), control the attachment and removal of O-GlcNAc on proteins, respectively. Whereas a variant OGT (mOGT) has been proposed as the main isoform that O-GlcNAcylates proteins in mitochondria, identification of a mitochondrial OGA has not been performed yet. Two splice variants of OGA (short and long isoforms) have been described previously. In this work, using cell fractionation experiments, we show that short-OGA is preferentially recovered in mitochondria-enriched fractions from HEK-293T cells and RAW 264.7 cells, as well as mouse embryonic fibroblasts. Moreover, fluorescent microscopy imaging confirmed that GFP-tagged short-OGA is addressed to mitochondria. In addition, using a Bioluminescence Resonance Energy Transfer (BRET)-based mitochondrial O-GlcNAcylation biosensor, we show that co-transfection of short-OGA markedly reduced O-GlcNAcylation of the biosensor, whereas long-OGA had no significant effect. Finally, using genetically encoded or chemical fluorescent mitochondrial probes, we show that short-OGA overexpression increases mitochondrial ROS levels, whereas long-OGA has no significant effect. Together, our work reveals that the short-OGA isoform is targeted to the mitochondria where it regulates ROS homoeostasis.

## 1. Introduction

O-GlcNAcylation is a post-translational modification corresponding to the attachment of a single O-linked N-acetylglucosamine (O-GlcNAc) to serine or threonine residues of cytosolic, nuclear, or mitochondrial proteins. This reversible modification regulates the localization, activity, and stability of proteins according to the nutritional environment of the cell, and, more specifically, according to glucose availability. Only two enzymes regulate O-GlcNAc level on proteins: OGT (O-linked N-Acetylglucosamine transferase), a glycosyl transferase that adds O-GlcNAc to proteins, and OGA, a β-N-Acetylglucosaminidase, distinct from acidic lysosomal hexosaminidase, which removes the O-GlcNAc from proteins. Numerous studies indicated a dynamic cross-talk between O-GlcNAcylation and phosphorylation, permitting fine-tuning of cell signaling pathways and regulation of gene expression [1,2]. O-GlcNAcylation has been involved in important human pathologies, including neurogenerative diseases, diabetes, and cancer [3].

Whereas protein O-GlcNAcylation in the cytosol and nucleus has been largely investigated, relatively little is known about O-GlcNAc cycling enzymes and their targets in the mitochondria. Alternative splicing of OGT results in the production of three different mRNA isoforms [4,5,6,7] that can code for three different proteins: a nucleo-cytoplasmic long form (ncOGT), a short isoform (sOGT) also found in the cytosol and nucleus, and, at least in humans and non-human primates, a mitochondria-targeted variant (mOGT) (Supplementary Figure S1). Several studies have shown the important role of O-GlcNAcylation in mitochondrial functions. Increases in O-GlcNAcylation of mitochondrial proteins have been observed upon high-glucose conditions [8,9], and recent work pointed to perturbation in the localization of mOGT in cardiomyocytes mitochondria from diabetic mice [10]. Several lines of evidence indicate that alteration of O-GlcNAc cycle disrupts mitochondrial homoeostasis [7,11,12,13,14,15,16], including alteration in reactive oxygen species production [14,16,17,18,19,20].

Biochemistry and proteomic studies have revealed that hundreds of mitochondrial proteins are O-GlcNAcylated, including key proteins involved in mitochondria bioenergetics, such as components of the respiratory chain complexes, ATP synthase, pyruvate dehydrogenase, Krebs cycle, and lipid metabolism enzymes [8,13,16,20,21,22,23,24]. Although it cannot be excluded that some of these proteins are O-GlcNAcylated in the cytosol prior to their export into mitochondria, several proteins encoded by mitochondrial DNA, such as MTCO1 (cytochrome oxidase 1), COX2 (cytochrome oxidase 2), and MT-ND4 (NADH:ubiquinone oxidoreductase core subunit 4), were also shown to be O-GlcNAcylated, confirming that O-GlcNAc cycling must occur within mitochondria [7]. The existence of an O-GlcNAc cycling machinery in the mitochondrial compartment is further indicated by the identification of the pyrimidine nucleotide carrier 1 as a UDP-GlcNAc transporter [10]. However, while OGA enzymatic activity has been demonstrated in mitochondria [8,10], the protein isoform involved has not been characterized. Alternative splicing of OGA also results in the production of two different mRNAs, coding for either long or short OGA isoforms [25,26]. The human long OGA comprises an O-GlcNAcase activity in its N-terminal side and a pseudo histone acetyltransferase (HAT) domain in its C-terminal side [26]. In the short OGA, the HAT domain is deleted, and a small-intronic-derived-sequence gives rise to a unique 15 amino acids C-terminal peptide. While the long OGA (L-OGA) isoform has been largely studied and was shown to be mainly cytoplasmic and nuclear, conflicting results have been reported concerning the short (S-OGA) isoform. Indeed, in glioblastoma cells, Comtesse et al. detected by Western blot a band of 75 kDa in the nuclear fraction that they assumed to be S-OGA [27]. In contrast, by fluorescence microscopy, Hanover’s group did not detect GFP-tagged S-OGA in the nucleus but rather suggested a lipid-droplet localization for this protein in HeLa cells [28].

Previous studies in cultured cells, animal tissues, and human samples have described a strong correlation between ncOGT and L-OGA expression [29,30,31,32], permitting tight control of O-GlcNAc level in the cell. While analyzing OGT and OGA mRNA expression levels in human leukocytes from healthy donors, we noticed that in contrast to L-OGA mRNA, which correlated with ncOGT mRNA, S-OGA mRNA expression did not correlate with ncOGT but was tightly correlated with the mitochondrial mOGT mRNA ( Supplementary Figure S1). This suggested a role for the short OGA isoform in the mitochondria and prompted us to evaluate its addressing to this organelle. We discovered that S-OGA is, indeed, the main OGA isoform in mitochondria and that it is involved in the control of ROS levels in this organelle.

## 2. Material and Methods

### 2.1. Antibodies

Anti-OGA antibody (NBP2-32233) was from Novus Biologicals (Littleton, USA). Anti-ATP-5A antibody (15H4C4) was from Abcam (Cambridge, UK). Anti-GFP antibody was from Roche (clones 7.1–13.1) (Indianapolis, USA). Anti-human GAPDH (sc-47724) and anti-alpha Tubulin (sc-8035) antibodies were from Santa Cruz (Dallas, USA).

### 2.2. Expression of S- and L-OGA mRNA in Human Leukocytes

Human leukocytes were obtained from blood samples of healthy volunteers (age 44.7 ± 1.7; 32 females, 35 males) from the French Blood Agency (Etablissement Français du Sang, Ile-de-France, Site Trinité; Agreement number INSERM-EFS:18/EFS/030). For each individual, 5–10 mL of blood were collected in EDTA tubes. Leucocytes were isolated after red blood cell lysis in 3 volumes of RBC Lysis Buffer (Santa Cruz). Leucocytes were pelleted by centrifugation at 280× *g* during 5 min. This procedure was repeated once or twice to eliminate residual red blood cells. The pellet was then washed in PBS, lysed in Trizol, and total RNA were isolated as described previously [33]. RT-qPCR were performed as described previously [32] using the primers indicated in Supplementary Table S1.

### 2.3. Preparation of Cytosolic and Mitochondrial-Enriched Fractions

Cells were cultured as described previously [34,35]. Preparation of mitochondrial-enriched fractions was performed as described below. HEK-293T cells and mouse embryonic fibroblasts (MEF) were cultured to confluence in T-175 flasks. Cells from one T-175 culture flask were collected by trypsin digestion, washed in PBS, and resuspended in 1 mL of ice-cold homogenization buffer containing 10 mM Hepes, pH 7.4, 250 mM sucrose, 1 mM AEBSF, and 1 mg/mL of pepstatin, antipain, leupeptin, and aprotinin. RAW264.7 cells were cultured to confluence in B-10 culture dishes. Cells from one B-10 dish were washed with PBS and then scratched in 1 mL of ice-cold homogenization buffer. Cells were then homogenized on ice by flushes (30 up and down) through a 1 mL syringe with a 22 G needle. Homogenates were then centrifuged twice at 1000× *g* for 10 min to pellet and discard nuclei and large debris. Mitochondria were then pelleted by centrifugation at 11,000× *g* during 10 min, washed with 1 mL homogenization buffer, centrifuged at 11,000× *g* for 10 min, and resuspended in the same buffer. The supernatant of the first 11,000× *g* centrifugation, mainly containing the cytosolic fraction, and the mitochondrial-enriched pellet fraction were then stored at −80 °C for subsequent analysis by Western blotting.

15–45 µg of proteins from either cytosolic or mitochondrial-enriched fractions were submitted to Western blotting as described previously [36]. Mitochondrial fractions were controlled with anti-ATP5A and cytosolic fractions with anti-human GAPDH or anti-alpha tubulin antibodies.

### 2.4. Confocal Microscopy Experiments

HEK-293T cells plated on polylysine-coated coverslips were transfected with cDNA (100 ng/40,000 cells) coding for mitochondrial-targeted mCherry (mCherry-Mito-7, a gift from Michael Davidson (Addgene plasmid # 55102) [37]) and either GFP-tagged long or short OGA isoforms (gifts from John A. Hanover). 48 h after transfection, cells were fixed with 4% paraformaldehyde and stained with DAPI (4′,6-diamidino-2-phenylindole) for visualization of the nuclei. Coverslips were sealed with ProLong diamond anti-fade mounting media (ThermoFisher Scientific, Waltham, USA) and analyzed by confocal microscopy. Confocal and structured illumination microscopy (SIM) were performed at the MicroPICell Facility of the University of Nantes using an inversed confocal Nikon A1 microscope coupled to the super resolution N-SIM. Z stack of 0.12 micron was performed using a 100× oil-immersion lens with high NA (SR ApoTIRF 100×, oil, NA: 1.49, Nikon, Tokyo, Japan). Images were acquired using NIS 4.2 software.

HEK-293T and HeLa cells plated on coverslips in a 6-well plate (3 × 10^5^ cells/well) were transfected with 10 ng of cDNA coding for GFP or GFP-IdP using lipofectamine 2000 (ThermoFisher Scientific). Cells were labeled with 200 nM MitoTracker (ThermoFisher Scientific) during 45 min at 37 °C and then fixed with 4% paraformaldehyde, stained with DAPI (4′,6-diamidino-2-phenylindole) for visualization of the nuclei, and analyzed by confocal microscopy using an inverted microscope (Leica DMI6000, objective lens 100×).

### 2.5. BRET Experiments

The coding sequence of mitochondrial targeting sequence from human COX8A was inserted upstream of the cDNA coding for the general O-GlcNAc-BRET biosensor described previously [35,38]. HEK-293T cells were co-transfected with this mitochondrial O-GlcNAc-BRET biosensor and either pcDNA3, S-OGA, or L-OGA plasmids. Cells transfected with luciferase alone and either pcDNA3, S-OGA, or L-OGA plasmids were used to correct for background signal. BRET experiments were then performed exactly as described previously [39] using the Tristar2 LB 942 plate reader (Berthold, Dieue-sur-Meuse, France). Briefly, cells were pre-incubated for 5 min in PBS in the presence of 5 µM coelenterazine. Each measurement corresponded to the signal emitted by the whole population of cells present in a well. BRET signal was expressed in milliBRET Unit (mBU). The BRET unit has been defined previously as the ratio 530 nm/485 nm obtained in cells expressing both luciferase and YFP (yellow fluorescent protein), corrected by the ratio 530 nm/485 nm obtained under the same experimental conditions in cells expressing only luciferase [40,41]. In each experiment, the mean of at least 10 repeated BRET measurements in a given experimental condition (see Supplementary Figure S4) was taken as the BRET value obtained in this experimental condition [35]. Delta BRET corresponded to the difference in BRET signal measured in cells transfected with pcDNA3 and cells transfected with either S-OGA or L-OGA.

### 2.6. Determination of Mitochondrial ROS Using MitoROS Probe

Mitochondrial ROS level was assessed using a Mitochondrial Superoxide Assay Kit according to the manufacturer’s instructions (Abcam). Briefly, HEK293-T cells (10^5^ cells/well in 12 well plates) were transfected with 500 ng of pcDNA3 or cDNA coding for S-OGA or L-OGA using Lipofectamine 2000. 24 h after transfection, cells were transferred into 96-well black microplate and cultured for an additional 24 h. Cells were then incubated with the MitoROS^TM^580 fluorescent probe (100 µL of MitoROS^TM^580 stain working solution added in each well) for 1 h at 37 °C. Fluorescence emission at 590 nm was then measured after stimulation at 540 nm using a CLARIOstar fluorimeter (BMG labtech, Orgenberg, Germany). Antimycin A was used as a positive control in these experiments, according to the manufacturer’s instructions (see Supplementary Figure S5A).

### 2.7. Determination of Mitochondrial H_2_O_2_ Using HyPer7 Fluorescent Probe

HyPer7 (pCS2+MLS-HyPer7, a gift from Vsevolod Belousov, Addgene, plasmid #136470)) is an ultrasensitive fluorescent ratiometric probe for detection of mitochondrial H_2_O_2_. This GFP probe has two excitation maxima at 400 and 499 nm and one emission peak centered at 516 nm. Upon oxidation, excitation and absorption spectra of Hyper7 changes in a ratiometric way with a decrease at 400 nm and an increase of the 499 nm peak [42].

HEK293-T cells (10^5^ cells/well in 12-well plates) were co-transfected with 500 ng of cDNA coding for HyPer7 and 500 ng of pcDNA3 or cDNA coding for S-OGA or L-OGA using Lipofectamine 2000. 24 h after transfection, cells were transferred into 96-well black microplate and cultured for an additional 24 h. Culture medium was removed and the cells were washed with PBS and incubated in 100 µL PBS for fluorescence determination. Fluorescence was measured at 515/40 nm after excitation at 390/22 nm and 485/15 nm using a LB942 Tristar2 Berthold fluorometer. After removing background fluorescence, the ratio of fluorescence emission after excitation at 485 nm to fluorescence emission after excitation at 390 nm was taken as relative measurement of mitochondrial H_2_O_2_ levels. Results were expressed as 485/390 ratio in pcDNA3, S-OGA or L-OGA transfected cells. In preliminary experiments, we verified that a dose-dependent effect of exogenous H_2_O_2_ on HyPer7 signal could be detected in HEK-293T cells (see Supplementary Figure S5B).

### 2.8. Statistical Analysis

Statistical analyses were performed using PRISM software. Comparisons between groups were performed using Student’s *t*-test, or ANOVA followed by Dunnett’s post-test for multiple comparison analysis. Correlations were performed using Pearson analysis.

## 3. Results

Long OGA has a theoretical molecular weight of 102 kDa but is well known to run on SDS-PAGE with an apparent molecular weight of 130 kDa [43]. To evaluate the migration profile of short OGA on SDS-PAGE, we first transfected HEK-293T cells with pcDNA3 or with plasmids coding for either S-OGA or L-OGA. Proteins from total cell lysates were submitted to SDS-PAGE followed by Western blotting using an antibody directed against a region common to both isoforms (residues 500–550, Novus Biologicals antibody). As shown in Supplementary Figure S2A, transfected S-OGA and L-OGA were readily detected in total cell lysate. As expected, L-OGA has an apparent molecular weight of about 130 kDa on SDS-PAGE. S-OGA, which has a theoretical molecular weight of 76 kDa, ran with an apparent molecular weight of about 95–100 kDa. Therefore, similar to L-OGA, S-OGA has an apparent molecular weight on SDS-PAGE higher than predicted from its amino-acid sequence.

**Figure 1:**
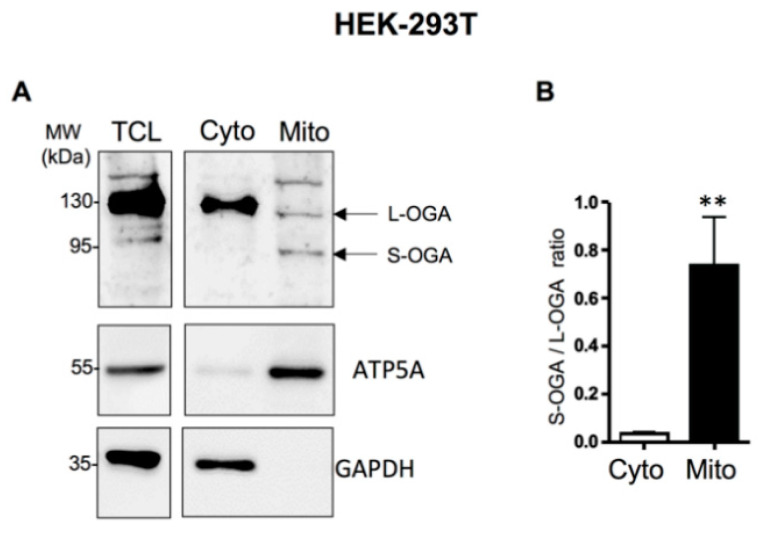

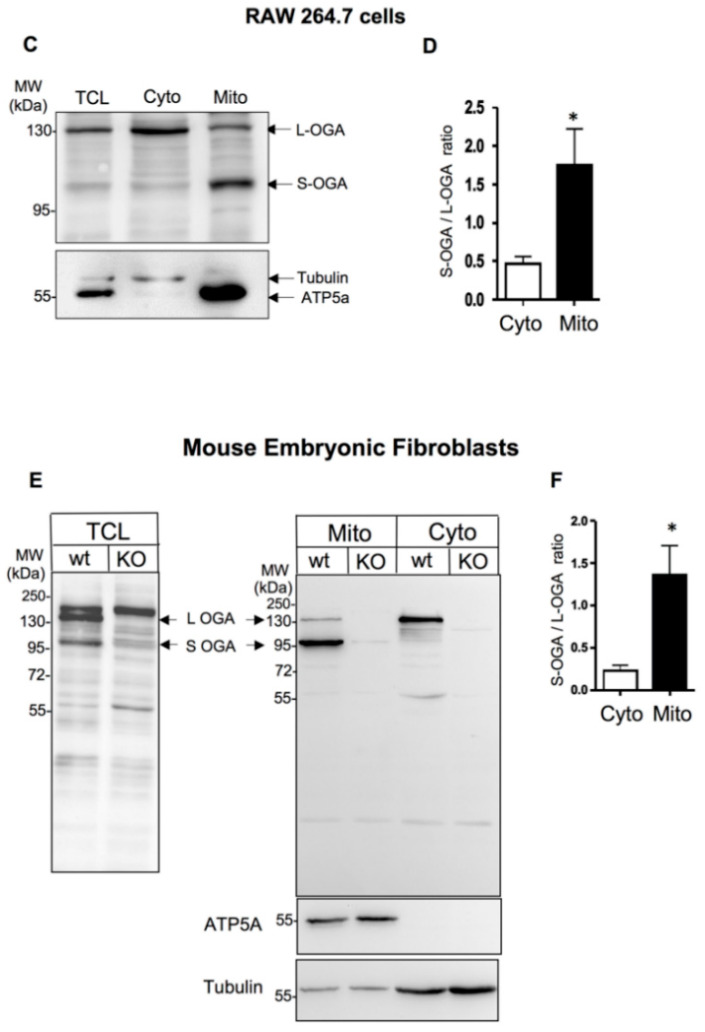
Detection of long and short OGA isoforms in HEK-293T cells and mouse embryonic fibroblasts (MEF). (**A**) Detection of endogenous S-OGA (~95 kDa) and L-OGA (~130 kDa) bands in total cell lysates (TCL), mitochondrial-enriched (Mito), and cytosolic-enriched (Cyto) fractions from HEK-293T cells by Western blotting with anti-OGA Novus antibody. Mitochondrial and cytosol fractions were controlled using anti-ATP5A and anti-GAPDH antibodies. The 95 kDa band was essentially recovered in the mitochondria-enriched fraction, whereas the 130 kDa band was mostly recovered in the cytosol-enriched fraction. (**B**) Densitometric analysis of the 95 kDa and 130 kDa OGA bands in HEK-293T cells. The results are the mean ± SEM of the ratio of the 95 kDa to the 130 kDa OGA bands detected in cytosol- and mitochondria-enriched fractions (*n* = 9; **: *p* < 0.01). (**C**) Detection of endogenous S-OGA (~95 kDa) and L-OGA (~130 kDa) bands in total cell lysates (TCL), mitochondria-enriched (Mito), and cytosol-enriched (Cyto) fractions from RAW 264.7 cells by Western blotting with anti-OGA Novus antibody. Mitochondrial and cytosol fractions were controlled using anti-ATP5A and anti-tubulin antibodies. The 95 kDa band was predominant in the mitochondria-enriched fraction, whereas the 130 kDa band was predominant in the cytosol-enriched fraction. (**D**) Densitometric analysis of the 95 kDa and 130 kDa OGA bands in RAW 264.7 cells. The results are the mean ± SEM of the ratio of the 95 kDa to the 130 kDa OGA bands detected in cytosol- and mitochondria-enriched fractions (*n* = 8; *: *p* < 0.05). (**E**) Detection of endogenous S-OGA (~95 kDa) and L-OGA (~130 kDa) bands in total cell lysates (TCL), mitochondria-enriched (Mito), and cytosol-enriched (Cyto) fractions from wild-type (wt) and OGA-KO mouse embryonic fibroblasts (MEF) by Western blotting with anti-OGA Novus antibody. Mitochondrial and cytosol fractions were controlled using anti-ATP5A and anti-tubulin antibodies. The 95 kDa band was predominant in the mitochondria-enriched fraction of wt-MEF, whereas the 130 kDa band was predominant in the cytosol-enriched fraction of wt-MEF. Both bands were absent in the corresponding fractions in OGA-KO MEF. An additional band (about 180 kDa) was also detected in total cell lysates, but this band was present in both wt and OGA-KO MEF, indicating that it corresponded to an unrelated protein. (**F**) Densitometric analysis of the 95 kDa and 130 kDa OGA bands in wt-MEF. The results are the mean ± SEM of the ratio of the 95 kDa to 130 kDa OGA signals detected in cytosol- and mitochondria-enriched fractions (*n* = 6; *: *p* < 0.05).

We then evaluated the relative distribution of endogenous L-OGA and S-OGA in total cell lysate (TCL)-, cytosolic (Cyto)-, and mitochondrial-enriched (Mito) fractions from HEK-293-T cells. In total cell lysates, we detected a major band of about 130 kDa corresponding to the expected molecular weight of the long OGA, and fainter bands, including a band of about 95 kDa, possibly corresponding to the short OGA isoform (Figure 1A). Cell fractionation experiments indicated that L-OGA was mainly recovered in the cytosolic fraction, whereas S-OGA was essentially recovered in the mitochondrial-enriched fraction, although a band corresponding to L-OGA and an additional band of higher molecular weight were also detected in this fraction. Densitometric analysis of the blots indicated that the relative amount of S-OGA over L-OGA was much higher in the mitochondrial-enriched fraction (Figure 1B). A similar result was obtained in mouse macrophage-derived RAW264.7 cells, indicating that S-OGA is also preferentially expressed in mitochondria-enriched fraction of rodent cell (Figure 1C,D).

Because antibodies quite often recognize non-specific bands that can be mistaken for the proteins of interest [6], we wanted to ensure that the 130 kDa and 95 kDa bands detected by Novus anti-OGA antibody indeed corresponded to OGA by using cytosol- and mitochondria-enriched fractions of embryonic fibroblasts (MEF) from wild-type and OGA-KO mice [44] (Figure 1E). These two bands were detected in total cell lysates from wt-MEF, but not in OGA-KO MEF (left blot). An additional band of about 180 kDa was also detected in total cell lysates, but it was present in both wt and OGA-KO MEF, indicating that it corresponded to an unrelated protein recognized by this antibody. Cell fractionation of MEF showed that S-OGA was clearly the predominant form in the mitochondria-enriched fraction while L-OGA was predominant in the cytosol-enriched fraction (Figure 1E,F). These bands were undetectable in the corresponding fractions from OGA-KO MEF, confirming the identity of the bands and the good specificity of the antibody.

To further establish the cellular localization of S-OGA and L-OGA, we transfected GFP-tagged versions of these proteins in HEK-293T cells (Supplementary Figure S2B). In agreement with Keembiyehetty et al. [28], transfected GFP-tagged S-OGA and L-OGA were detected with an anti-GFP antibody as bands of about 130 kDa and 160 kDa, respectively, as expected for proteins of about 95 and 130 kDa fused with the 28 kDa GFP protein. As shown in Supplementary Figure S2B, GFP-tagged L-OGA was more abundant than S-OGA-GFP in the cytosolic-enriched fraction. In contrast, GFP-tagged S-OGA was more abundant than GFP-tagged L-OGA in the mitochondria-enriched fraction, despite higher expression of GFP-tagged L-OGA in total cell lysates, confirming the preferential addressing of S-OGA in the mitochondria (Supplementary Figure S2B,C). Preferential localization of S-OGA in the mitochondria-enriched fraction was even more obvious when HEK-293T cells were co-transfected with cDNA coding for GFP-tagged S-OGA and L-OGA. In these cells where both S- and L-OGA were overexpressed, the mitochondria-enriched fraction contains mainly GFP-tagged S-OGA, whereas GFP-tagged L-OGA was much more abundant in the cytosolic fraction (Supplementary Figure S2D,E). Preferential recovery of GFP-tagged S-OGA in mitochondrial fraction was also observed in MEF transfected with these constructs (Supplementary Figure S2F,G).

**Figure 2:**
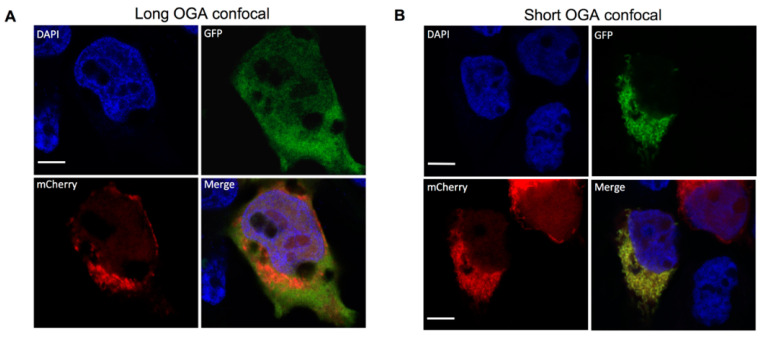

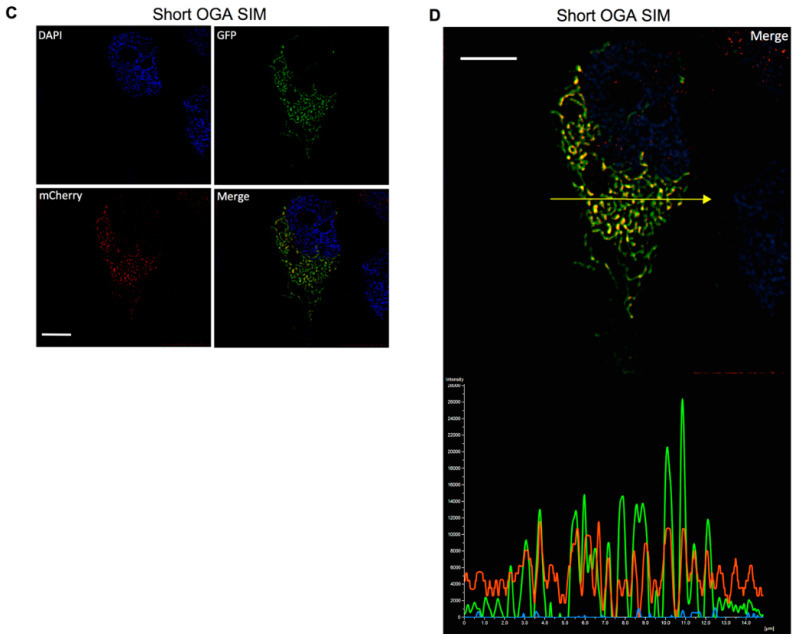
Detection of GFP-tagged long and short OGA isoforms in HEK-293T cells. HEK-293T cells were transfected with mitochondrial-targeted mCherry and either GFP-tagged short or long OGA isoforms, fixed, stained with DAPI for visualization of the nuclei (blue), and analyzed by confocal microscopy in the GFP (green) and mCherry (red) channels. Merge images are shown. (**A**) The long OGA isoform was detected in the cytosol and the nucleus but poorly co-localized with mito-cherry labeled mitochondria. (**B**) Co-localization of the short OGA isoform with mito-mCherry in a cell expressing GFP-tagged short OGA. (**C**,**D**) Structured illumination microscopy (SIM) confirmed localization of S-OGA in the mitochondria. (**C**) Separated SIM and merge images of this cell in the GFP, mCherry, and Dapi channels are shown. (**D**) Densitometric analysis of the different signals detected along the arrow shown on the upper panel indicates co-localization of S-OGA-GFP with mito-mCherry (green: S-OGA-GFP, red: mito-mCherry, blue: Dapi staining of the nucleus, white bar scale: 5 μm). Representative images are shown.

Although S-OGA is clearly preferentially recovered in the mitochondrial-enriched fractions, both S-OGA and L-OGA isoforms were detected in cytosolic and mitochondrial fractions. Nonetheless, mitochondrial- and cytosol-enriched fractions may have cross-contaminated each other during the centrifugation procedure, resulting in the recovery of S-OGA in the cytosolic fraction and L-OGA in the mitochondrial fraction. To confirm specific localization of S-OGA in the mitochondria, we used confocal microscopy. Since no antibodies are available to specifically detect the short OGA isoform by cell imaging, we co-transfected HEK-293T cells with cDNAs coding for either GFP-tagged short or long OGA isoforms and a mitochondrial-targeted mCherry protein. Confocal microscopy imaging (Figure 2) revealed that L-OGA isoform was diffusively found in the cytosol and the nucleus (Figure 2A), whereas S-OGA was not detected in the nucleus (Figure 2B), in agreement with Keembiyehetty et al.’s observation [28]. In contrast, S-OGA isoform co-localized with mitochondrial mCherry protein (Figure 2B), indicating specific mitochondrial addressing of this isoform. Mitochondrial localization of the short OGA isoform was confirmed using structured illumination microscopy [45] (Figure 2C,D).

**Figure 3:**
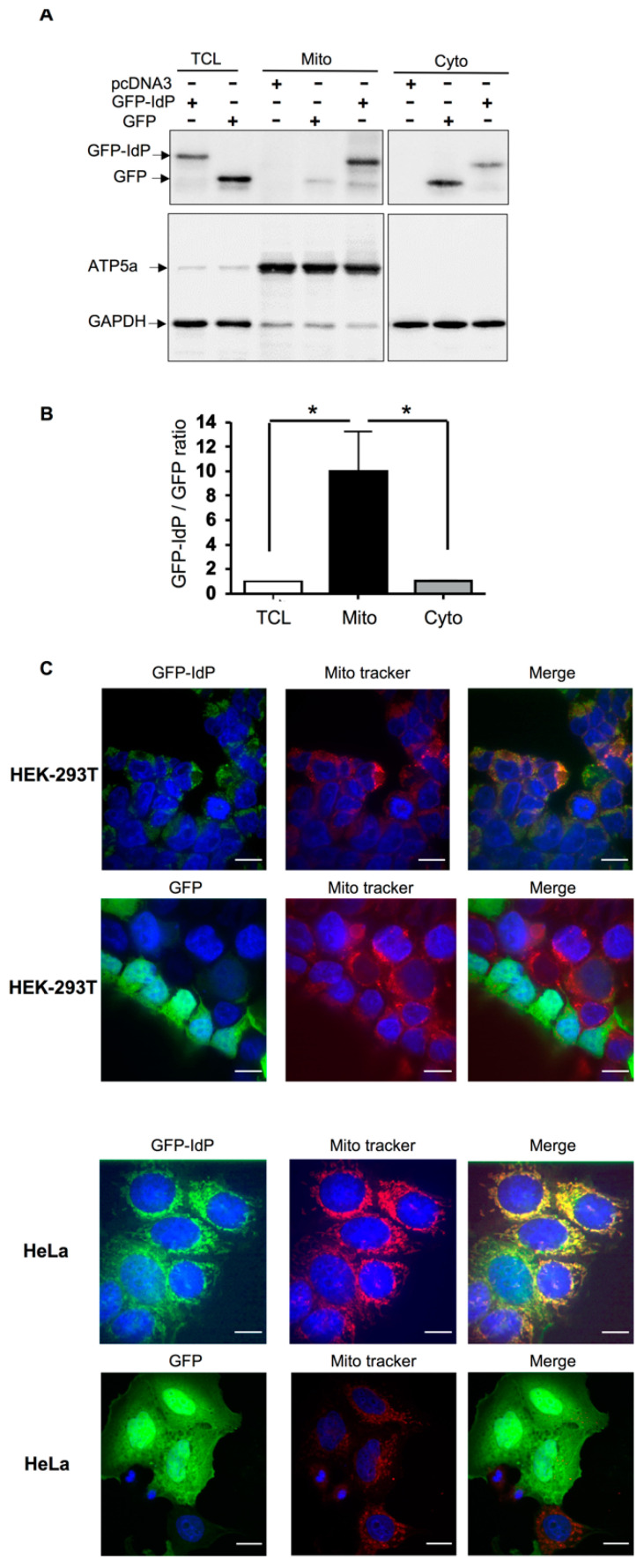
The intron-derived peptide of short OGA is sufficient to target GFP to the mitochondria. (**A**) HEK-293T cells were transfected with cDNA coding for either GFP or GFP fused at its C-terminus with OGA intron-derived peptide (GFP-IdP). Total cell lysate, mitochondrial-, and cytosol-enriched fractions were analyzed by Western blotting with anti-GFP antibody. GFP-IdP was more abundant in the mitochondrial-enriched fraction whereas GFP was more abundant in the cytosol-enriched fraction. (**B**) Densitometric analysis of the GFP and GFP-IdP bands. The results are the mean ± SEM of the ratio of the GFP-IdP to GFP signals detected in TCL, cytosol-, and mitochondria-enriched fractions (*n* = 5; *: *p* < 0.05). (**C**) HEK-293T cells and HeLa cells were transfected with cDNA coding for GFP or GFP fused at its C-terminus with OGA intron-derived peptide (GFP-IdP), fixed, and labeled with mitotracker. Confocal microscopy experiments confirmed co-localization of GFP-IdP with mitotracker. GFP alone was widely distributed into the cells, including the nucleus.

S-OGA results from an mRNA splicing that retains an intronic sequence coding for a 15-amino-acid peptide located in the C-terminus of the human short OGA isoform. This peptide sequence, which is not present in L-OGA, is fully conserved in five primate species and partially conserved in other mammalian species (Supplementary Figure S3). We hypothesized that this sequence may serve as a mitochondria-targeting sequence. To test this hypothesis, we introduced the sequence coding for this intron-derived peptide (IdP) at the C-terminus of the GFP. Transfection of HEK-293T cells with cDNA coding for either GFP or GFP-IdP showed that GFP-IdP was essentially recovered in the mitochondria -enriched fraction, in contrast to GFP alone, which was essentially recovered in the cytosolic fraction (Figure 3A,B). Confocal microscopy experiments confirmed mitochondrial localization of GFP-IdP in HEK-293T as well as in HeLa cells (Figure 3C), whereas GFP was uniformly distributed into the cell. This result suggests that the intron-derived peptide may act as a mitochondria-addressing sequence for S-OGA.

**Figure 4:**
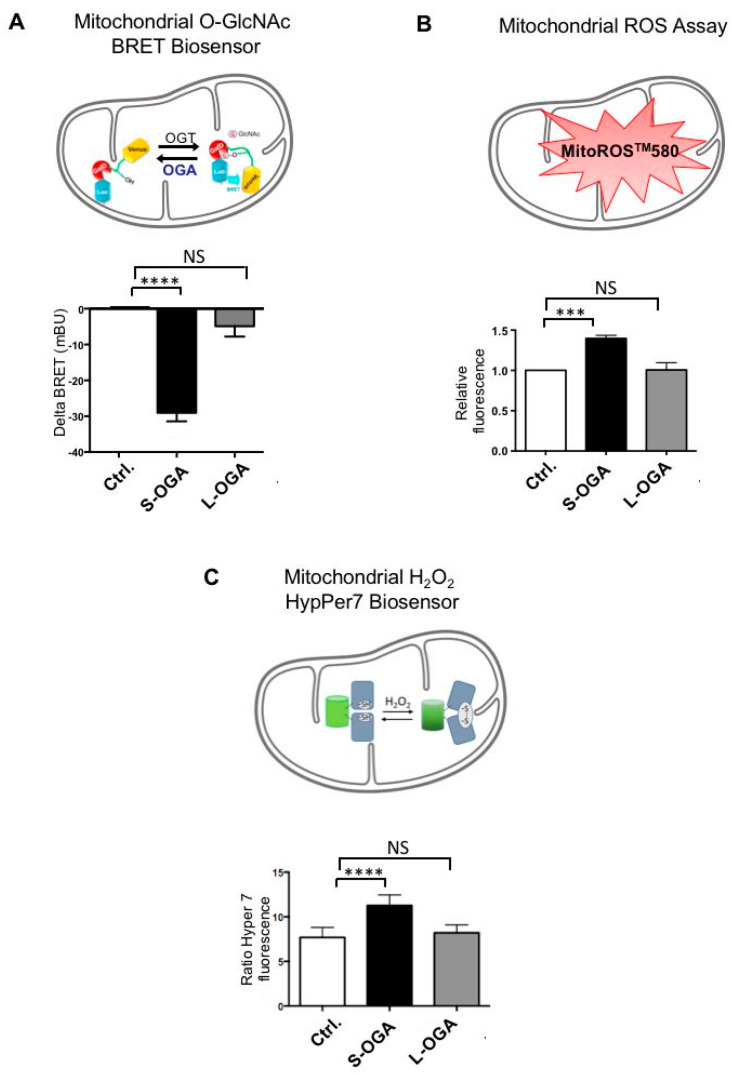
Short OGA expression reduced O-GlcNAcylation and increased ROS levels in the mitochondria. (**A**) HEK-293T cells were co-transfected with the mitochondria-targeted BRET biosensor and either pcDNA3, S-OGA, or L-OGA. The graph shows the difference in BRET signal (delta BRET, expressed in milliBRET units) between pcDNA3 transfected cells and either S-OGA or L-OGA transfected cells (*n* = 9; ****: *p* < 0.0001, NS: non-significative). S-OGA markedly reduced BRET signal, indicating decreased O-GlcNAcylation of the mitochondrial O-GlcNAc biosensor by this isoform. (**B**) HEK-293T cells transfected with pcDNA3, S-OGA, or L-OGA were incubated for 1 h with MitoROS^TM^580. Fluorescence emission at 590 nm was then measured after excitation at 540 nm. Results are expressed as fluorescence in S-OGA or L-OGA transfected cells relative to pcDNA3 transfected cells (*n* = 5; ***: *p* < 0.001; NS: non-significative). (**C**) HEK-293T cells were co-transfected with the mitochondria H_2_O_2_ biosensor (HyPer7) and either pcDNA3, S-OGA, or L-OGA. In each experiment, the ratio of fluorescence emission at 516 nm after excitation at 499 nm to fluorescence emission at 516 nm after excitation at 400 nm was determined. Results are expressed as 499/400 ratio in pcDNA3, S-OGA, or L-OGA transfected cells (*n* = 13, ****: *p* < 0.0001, NS: non-significative).

To determine whether S-OGA expression can regulate protein O-GlcNAcylation in mitochondria, we used a BRET-based O-GlcNAc biosensor that monitors O-GlcNAcylation activity in living cells [35,38] (Supplementary Figure S4). To specifically monitor O-GlcNAcylation in mitochondria, the mitochondrial targeting sequence of cytochrome oxidase subunit 8A (COX8A) was fused to the cDNA coding for the BRET-O-GlcNAc biosensor (Mitochondrial O-GlcNAc BRET biosensor, Supplementary Figure S4). HEK293-T cells were co-transfected with this biosensor and either pcDNA3, S-OGA, or L-OGA. We observed that co-transfection of S-OGA markedly reduced basal BRET, whereas L-OGA had no significant effect on BRET signal (Figure 4A and Figure S4). These results strongly suggest that S-OGA is indeed an important regulator of protein O-GlcNAcylation level in the mitochondria.

Several studies have indicated that O-GlcNAcylation is involved in the regulation of oxidative stress [46]. Since mitochondria is an important player in ROS production, we evaluated the effect of S-OGA overexpression on mitochondrial ROS levels in HEK-293T cells. Using a mitochondrial superoxide detection assay (MitoROS^TM^580), we observed that S-OGA overexpression in HEK-293T cells significantly increased mitochondrial ROS level, when compared to pcDNA3 transfected cells, whereas L-OGA had no significant effect (Figure 4B).

In the mitochondria, ROS are readily converted into more stable and less toxic hydrogen peroxide H_2_O_2_ by superoxide dismutase [47]. HEK-293T cells were co-transfected with pcDNA3, S-OGA, or L-OGA, and a mitochondrial GFP-derived H_2_O_2_ biosensor (HyPer7 [42]), which permits to specifically monitor H_2_O_2_ level in the mitochondria. We observed that S-OGA expression resulted in a significant increase in H_2_O_2_ in the mitochondria, whereas L-OGA had no significant effect (Figure 4C).

Together, our results indicate that S-OGA in the mitochondria regulates protein O-GlcNAcylation, as well as superoxide and hydrogen peroxide levels in mitochondria.

## 4. Discussion

In the present work, we provided several lines of evidence indicating that S-OGA is a mitochondrial targeted isoform: (i) endogenous S-OGA is preferentially recovered as a 95–100 kDa band in mitochondria-enriched fraction of HEK-293T cells, RAW264.7 macrophages, and mouse embryonic fibroblasts; (ii) transfection of GFP-tagged OGA confirmed preferential recovery of S-OGA in the mitochondria-enriched fraction of HEK-293T and MEF; (iii) confocal microscopy experiments indicated that whereas L-OGA is detected in the cytosol and nucleus, S-OGA is preferentially addressed to the mitochondria; (iv) the C-terminal intron-derived sequence, specific to S-OGA, is sufficient to address GFP at the mitochondria in HEK-293T and HeLa cells; (v) S-OGA markedly reduced BRET signal produced by a mitochondria-targeted O-GlcNAc biosensor, whereas L-OGA does not; (vi) S-OGA, but not L-OGA, markedly increases mitochondrial ROS production.

Comtesse et al. [27] previously reported that S-OGA was localized in the nucleus fraction of a human glioblastoma cell line, on the basis of a band detected by Western blot at a molecular weight of 75 kDa using an in-house-made polyclonal antibody. However, in absence of any validation of this result using siRNA-treated or MEF-KO cells depleted of the protein of interest, caution should be taken concerning the specificity of the band detected by this antibody [6]. In addition, in the same experiment, these authors failed to detect L-OGA in the cytoplasm fraction, which, on their Western blot, was detected essentially in a membrane/cytoskeleton fraction, casting further doubt on the specificity of this antibody. Moreover, our transfection experiments with the cDNA coding for human OGA clearly indicated that S-OGA migrates at an apparent molecular weight of 95–100 kDa. In agreement with our observation, Li et al. observed that recombinant S-OGA migrated at a molecular weight of 97 kDa [48]. Finally, L-OGA has been shown to possess a caspase cleavage site that results in a fragment migrating at a molecular weight of about 70 kDa on SDS-PAGE [49,50]. Therefore, it is also possible that the signal detected around this molecular weight corresponds to a cleavage product of L-OGA [48]. These observations suggest that the band detected at 75 kDa by Comtesse et al. may correspond either to a non-specific signal detected by their antibody, or to a cleavage product of L-OGA.

In agreement with Keembiyehetty et al. [28], our confocal experiments indicated that L-OGA located diffusely in the cytosol and the nucleus, whereas S-OGA seems to be excluded from the nucleus. Our Western blotting and confocal microscopy experiments indicated that S-OGA is essentially addressed to the mitochondria, although other cellular localization cannot be excluded. Interestingly, Keembiyehetty et al. [28] found that S-OGA was localized associated with nascent lipid droplets in HeLa cells incubated with oleate. These authors proposed that the short OGA isoform participates in the regulation of lipid droplet assembly and mobilization. Numerous studies indicate physical as well as functional interactions between mitochondria and lipid droplets, facilitating lipid droplet mobilization and fatty acid oxidation [51,52,53,54]. Our observation that S-OGA is found associated with mitochondria suggests that this isoform may participate in the regulation of mitochondria–lipid droplet dialogue. This point certainly deserves further investigations.

Various studies have shown that perturbations of O-GlcNAc cycling that increase or decrease O-GlcNAcylation can affect mitochondria homoeostasis, respiration, and ROS production [7,11,12,13,14,15,16,17,18,19,20]. However, only few studies directly evaluated the specific effect of modulating the expression of mOGT on ROS production. Sacoman et al. [7] found that in HeLa cells, mOGT siRNA markedly affected mitochondrial homeostasis, including decreased mitochondrial content associated with a compensatory increase in oxygen consumption per mitochondrion, although without altering mitochondrial ROS production. More recently, Jozwiak et al. [16] showed that overexpression of mOGT increases mitochondrial ROS production. In the present work, we showed that overexpression of S-OGA also increases mitochondrial ROS production. Therefore, perturbation of the equilibrium between OGT and OGA in the mitochondria, by increasing the expression of one or the other enzyme, results in an increase in mitochondrial ROS production. This could correspond to an extension to the mitochondrial compartment of the emerging concept that O-GlcNAc level needs to be maintained within an optimal zone to preserve cellular homeostasis [55]. The mechanisms by which protein O-GlcNAcylation may affect mitochondrial ROS levels remains elusive, although several potential mechanisms associated with O-GlcNAcylation of specific subunit of the respiratory chain complexes and Krebs cycle enzymes have been proposed, including modulation of reverse electron transfer through respiratory chain complexes, alteration of mitochondrial transmembrane potential, and changes in mitochondrial respiration rate [14,16,20]. Alternatively, intramitochondrial O-GlcNAcylation could regulate ROS levels through modulation of intramitochondrial detoxifying enzymes, such as superoxide dismutase and peroxiredoxin 3, which were found to be O-GlcNAcylated in proteomics analysis [13,23]. Clearly, further extensive work is still needed to elucidate the molecular mechanisms by which O-GlcNAcylation regulates mitochondrial ROS.

In conclusion, in this work, we discovered that S-OGA is preferentially targeted to the mitochondria, where it appears to modulate ROS levels. Mitochondria is believed to be the main source of cellular ROS. Whereas H_2_O_2_ can act as a signaling molecule in the cell, excess ROS production and elevated H_2_O_2_ levels have deleterious effects and are involved in several pathological conditions, including cancer, inflammatory diseases, Type 2 diabetes, neurodegenerative diseases, and aging. The use of mitochondria-targeted small molecules has been proposed as a potential therapeutic strategy, most notably to specifically deliver antioxidants to this compartment [56,57]. The discovery that S-OGA is addressed to the mitochondria, and that it plays a role in the regulation of ROS levels in this organelle, may open new avenues for the development of molecules with potential therapeutic value. To this aim, it will be necessary to fully characterize S-OGA targets in the mitochondria, to elucidate its mechanism of action in the regulation of ROS production, and to develop molecules that specifically target S-OGA activity and/or interaction with its mitochondrial protein partners.

## Data Availability

Not applicable.

## Supplementary Materials

The following supporting information can be downloaded at: https://www.mdpi.com/article/10.3390/cells11111827/s1. Figure S1: Correlations between S-OGA and mOGT mRNA expression in human leukocytes, Figure S2: Expression of transfected S-OGA and L-OGA in HEK-293 T and mouse embryonic fibroblasts, Figure S3: Alignment of the sequences of short OGA intron-derived peptide in different mammalian species, Figure S4: Monitoring O-GlcNAcylation in mitochondria using the O-GlcNAc BRET biosensor, Figure S5: Effects of Antimycin A and H2O2 on MitoROS and HyPer7 fluorescent probes. Table S1: Sequences of oligonucleotides used for RT-qPCR.
