# Supplementary material for: Short O-GlcNAcase Is Targeted to the Mitochondria and Regulates Mitochondrial Reactive Oxygen Species Level
Source: Cells. 2022 Jun 2;11(11):1827. doi: 10.3390/cells11111827 (PMC9180253; doi:10.3390/cells11111827)

Supplementary Figure S1: Correlations between S-OGA and mOGT mRNA expression in human leukocytes (A) Alternative splicing of OGA results in the production of two different mRNAs, coding for either long or short OGA isoforms. The short mRNA isoform lacks the sequence coding for exons 11 to 16 but retains part of intron 10 as a coding sequence. The black box indicates the localization of the sequence that is specific for short OGA. 2 couples of primers were designed to specifically quantify mRNA expression of each isoform. (B) Alternative splicing of OGT results in the production of three different mRNAs, which can code for 3 different proteins: the two nucleo-cytoplasmic long (ncOGT) and short OGT variants (sOGT), and at least in human and other primates, a mitochondria-targeted variant (mOGT). The mOGT mRNA isoform is generated by the use of intron 4 as an alternative exon (exon 5). This transcript contains a unique ATG that produces a shorter isoform which comprises a 20 amino acid mitochondrial targeting sequence. 2 couples of primers were designed to specifically quantify mRNA expression of ncOGT and mOGT isoforms (the absence of any sequence specific to sOGT impairs evaluation of its expression by RT-qPCR). (C) Correlations between OGT and OGA mRNA splice variants were evaluated using Pearson's analysis. OGA mRNA expression levels were measured in leucocytes from healthy donors by quantitative RT-PCR and normalized to the expression of cyclophilin A mRNA. Expression levels of ncOGT correlated with long OGA mRNA (left panel) but not with short OGA mRNA (middle panel), while mOGT mRNA expression levels correlated with short OGA (right panel).

A

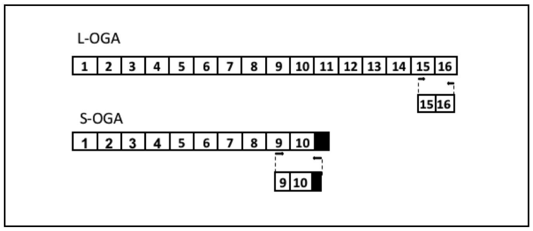

B

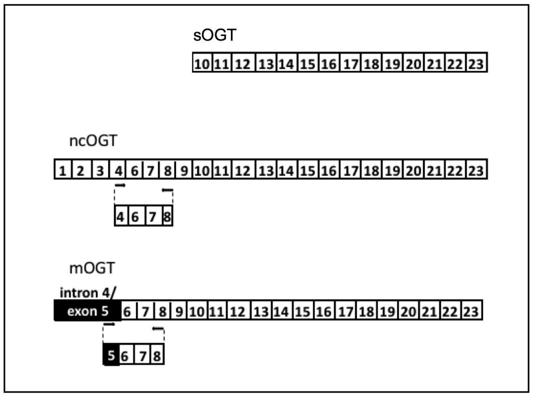

C

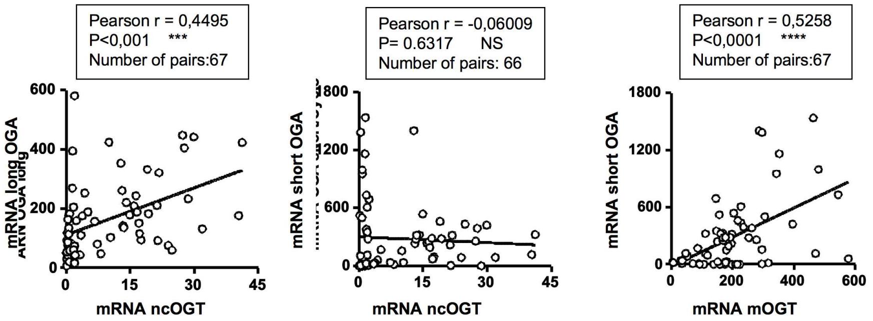

Supplementary Figure S2: Expression of transfected S-OGA and L-OGA in HEK-293T and mouse embryonic fibroblasts (A) HEK-293T cells were transfected with pcDNA3 empty vector or plasmids coding for S-OGA or L-OGA. S-OGA and L-OGA in total cell lysates were detected as bands with apparent molecular weights of about 95 kDa and 130 kDa by western-blotting using anti-OGA Novus antibody. (B) HEK-293T cells were transfected with plasmids coding for GFP-tagged S-OGA or L-OGA. GFP-tagged S-OGA and L-OGA (left panel) were detected in total cell lysates (TCL) as bands with apparent molecular weights of ~130 kDa and ~160 kDa by western-blotting with an anti-GFP antibody. In mitochondria-enriched fractions from these cells, recovery of transfected S-OGA was higher than L-OGA, whereas L-OGA recovery was higher in the cytosolic enriched fraction. Mitochondrial and cytosol fractions were controlled using anti-ATP5A and anti-GAPDH antibodies. (C) Densitometric analysis of the 130 kDa and 160 kDa GFP-tagged OGA bands in HEK-293T cells. The results are the mean ± SEM of the ratio of the 130 kDa to 160 kDa GFP-tagged OGA bands detected in cytosol- and mitochondria-enriched fractions (n=3; *: $p<0.05$). (D) HEK-293T cells were co-transfected with both plasmids coding for GFP-tagged S-OGA or L-OGA. GFP-tagged S-OGA and L-OGA (left panel) were detected in total cell lysates (TCL) as bands of apparent molecular weights of ~130 kDa and ~160 kDa by western-blotting with an anti-GFP antibody. In mitochondria-enriched fractions from these cells, recovery of transfected GFP-tagged S-OGA was much higher than GFP-tagged L-OGA, whereas GFP-tagged L-OGA was essentially recovered in the cytosolic enriched fraction. Mitochondrial and cytosol fractions were controlled using anti-ATP5A and anti-tubulin antibodies. (E) Densitometric analysis of the 130 kDa and 160 kDa bands in HEK-293T cells co-transfected with GFP-tagged S-OGA and L-OGA cDNAs. The results are the mean ± SEM of the ratio of the 130 kDa to 160 kDa GFP-tagged OGA signals detected in cytosol- and mitochondria-enriched fractions (n=3; *: $p<0.05$). (F) MEF were transfected with cDNA coding for either GFP alone, GFP-tagged S-OGA (130 kDa) or GFP-tagged L-OGA (160 kDa). Total cell lysate (TCL), mitochondria (Mito) and cytosolic (Cyto) enriched fractions from these cells were submitted to western-blotting using an anti-GFP antibody. Mitochondrial and cytosol enrichment was controlled using anti-ATP5A and anti-alpha tubulin antibodies. In mitochondria-enriched fractions, recovery of transfected GFP-tagged S-OGA was higher than GFP-tagged L-OGA, whereas GFP-tagged L-OGA recovery was higher in the cytosolic enriched fraction. (G) Densitometric analysis of the 130 kDa and 160 kDa GFP-tagged OGA bands in HEK-293T cells. The results are the mean ± SEM of the ratio of the 130 kDa to 160 kDa GFP-tagged OGA signals detected in TCL, cytosol- and mitochondria-enriched fractions (n=3; *: $p<0.05$).

Supplementary Figure S2

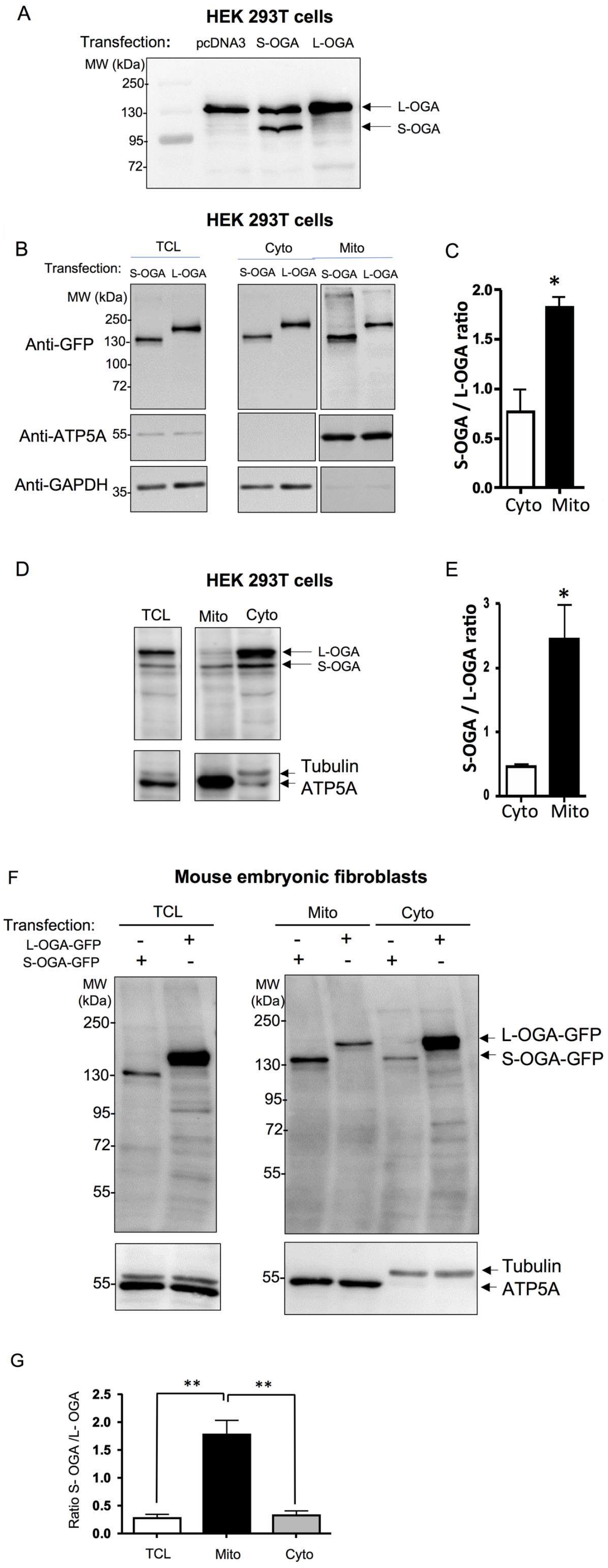

Supplementary Figure S3: Alignment of the sequences of short OGA intron-derived peptide in different mammalian species.

RCTRNNLFSSNILSL STOP	Human OGA
RCTRNNLFSSNILSL STOP	Gorilla OGA
RCTRNNLFSSNILSL STOP	Orangutan OGA
RCTRNNLFSSNILSL STOP	Chimpanzee OGA
RCTRNNLFSSNILSL STOP	Macaca mulatta OGA
RCTRNNLFSSNILSLKKKIIPQGEKHLGNWHFYKSFPFFA STOP	Marmoset OGA
RCTRNNLFSSDILSLKKKKIIP STOP	Pig OGA
RCTRNNLFSSDILSPKNEKKLSHREKNI STOP	Dog OGA
RCTRNNLFSDILSLKRKKKLSHREKNVYEISEFPTVFKFLA STOP	Goat OGA
RCTRNNLFSDILSLKRKKNYPTGRKTFRELVNFQLCLNSLPRFQRCSCCYRLEVQASVNITSTLIFLPWANSA STOP	Cow OGA
RCTSSSLLSSIYIVL STOP	Mouse OGA
RCYGSSSGFEQTVVLSVSIAKGKTLKK STOP	Rat OGA

Supplementary Figure S4: Monitoring O-GlcNAcylation in mitochondria using the O-GlcNAc BRET biosensor (A) The O-GlcNAc BRET biosensor is composed of Rluc8 luciferase fused to a lectin domain (GafD), a known OGT substrate peptide derived from casein kinase II, followed by the Venus variant of the yellow fluorescent protein. The human COX8A (Cytochrome Oxydase subunit 8A) pre-sequence was inserted upstream of this biosensor for specific targeting to the mitochondria. Basal BRET signal results from the balance between OGT and OGA activities. O-GlcNAcylation of CKII promotes its binding to GafD, resulting in an increased in BRET signal, whereas removal of the GlcNAc by OGA decreases binding of casein kinase II peptide to GafD lectin domain, resulting in a decrease in BRET signal. (B) Typical experiment showing the monitoring of BRET signal during 10 minutes in pcDNA3, S-OGA and L-OGA transfected HEK-293T cells. BRET signal was markedly decreased by S-OGA but barely affected by L-OGA.

A

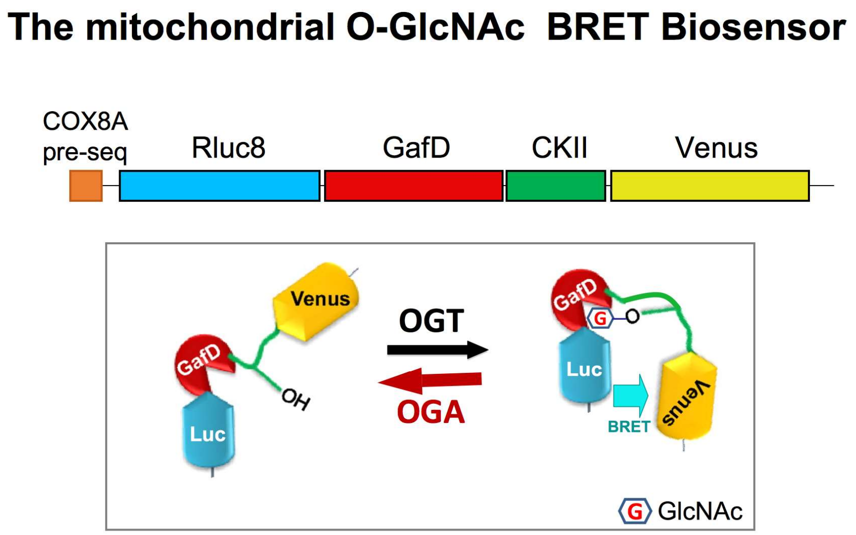

B

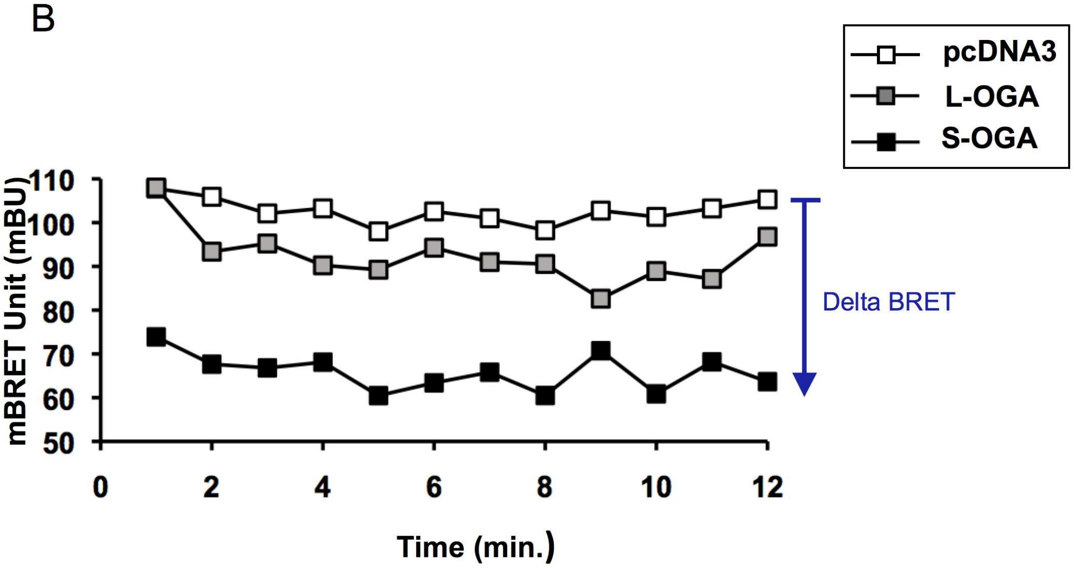

Supplementary Figure S5: Effects of Antimycin A and H_2O_2 on MitoROS and HyPer7 fluorescent probes

(A) Effect of Antimycin A on MitoROS™580 signal. HEK-293T cells were pre-incubated in absence or presence of Antimycin A (50µM) during 30 min and then labelled with MitoROS™580 signal for 1h. Fluorescence emission at 590 nm was then measured after excitation at 540 nm. Results are expressed as relative fluorescence of Antimycin A-treated to non-treated cells and are the mean ± SEM of 3 independent experiments. Background fluorescence was evaluated by measuring autofluorescence in non-labelled cells. (B) Dose-dependent effect of H_2O_2 on HyPer7 signal. HEK-293T cells transfected with the mitochondria H_2O_2 biosensor (HyPer7) were treated or not with 50, 100, or 200µM H_2O_2 for 1 min prior measurement of HyPer7 fluorescence as described in the Method section. Results are mean ± SEM of two independent experiments.

A

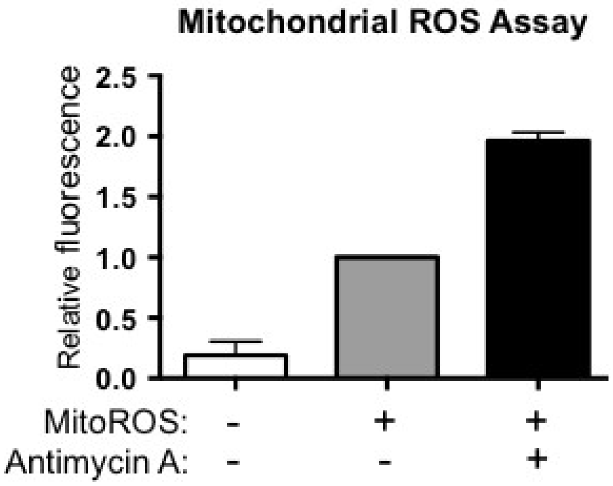

B

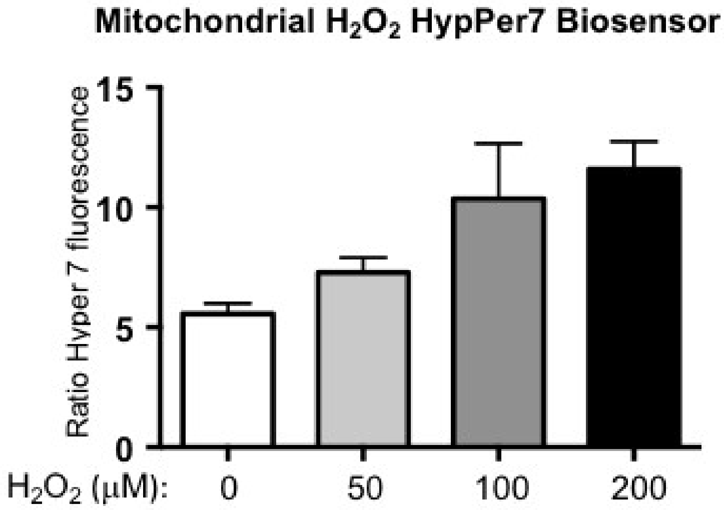

Supplement: Supplementary file 1 [file cells-11-01827-s001.zip › cells-1716428-supplementary.pdf]
